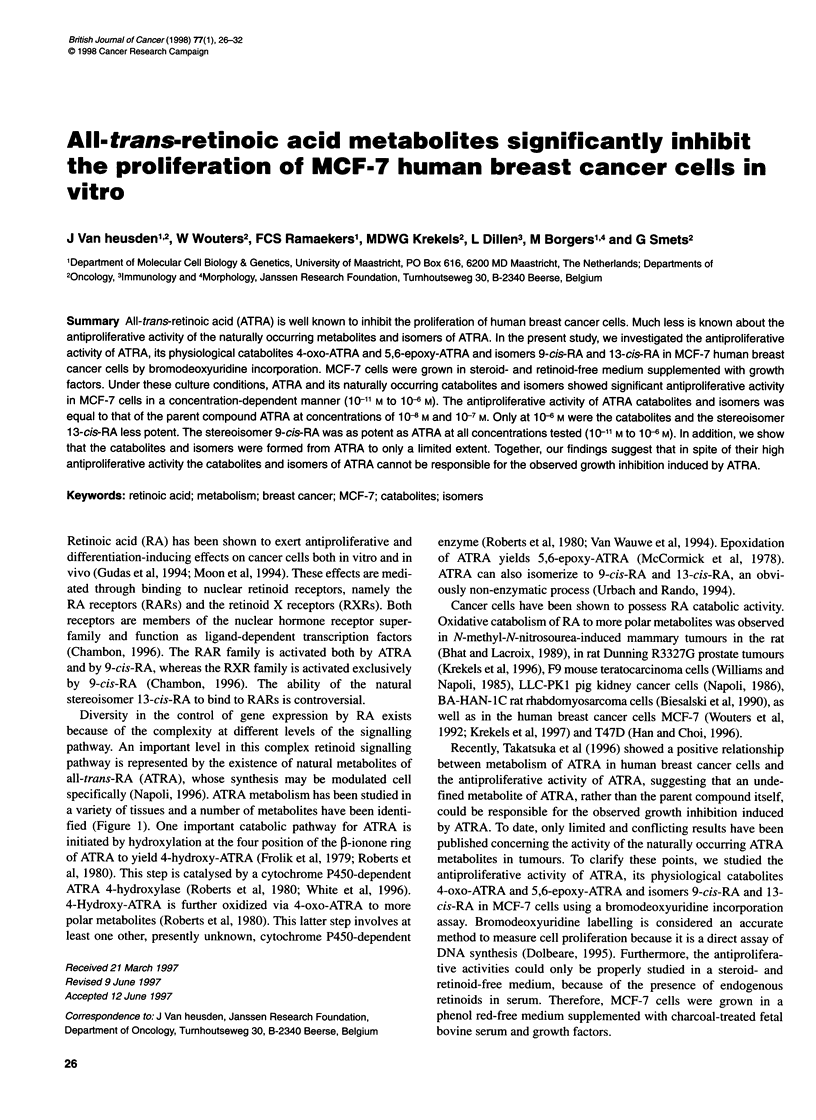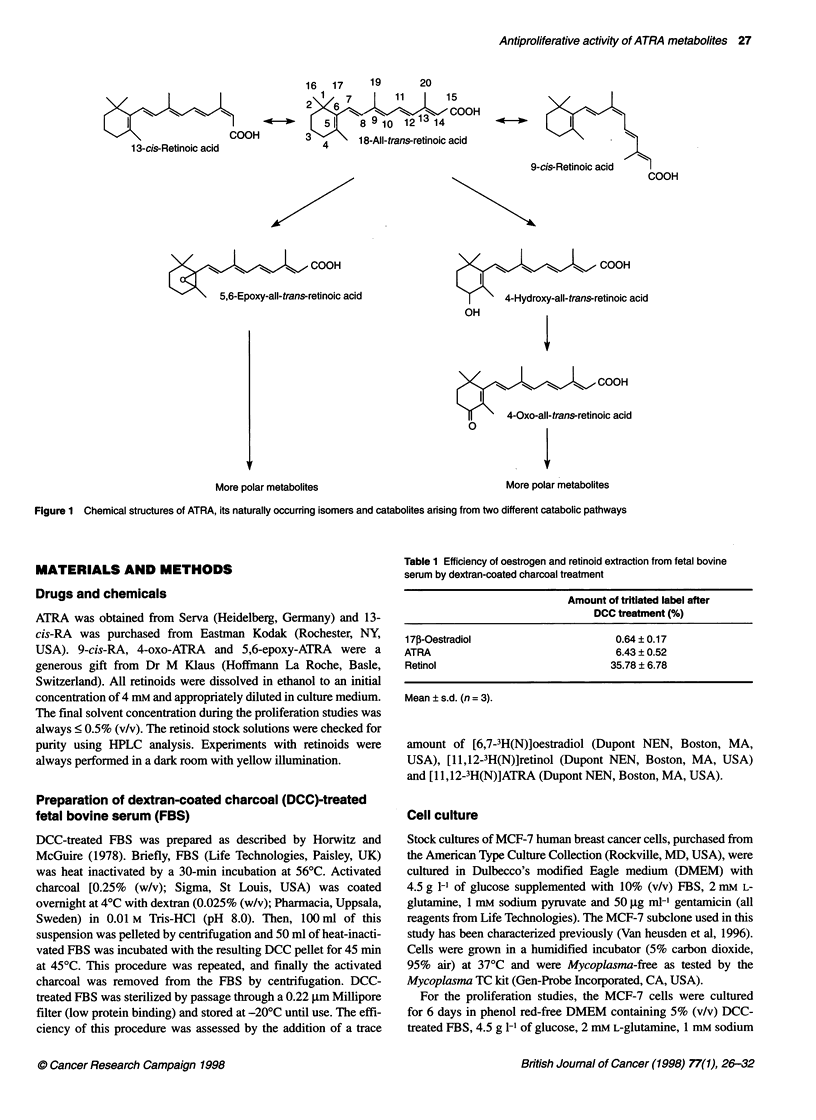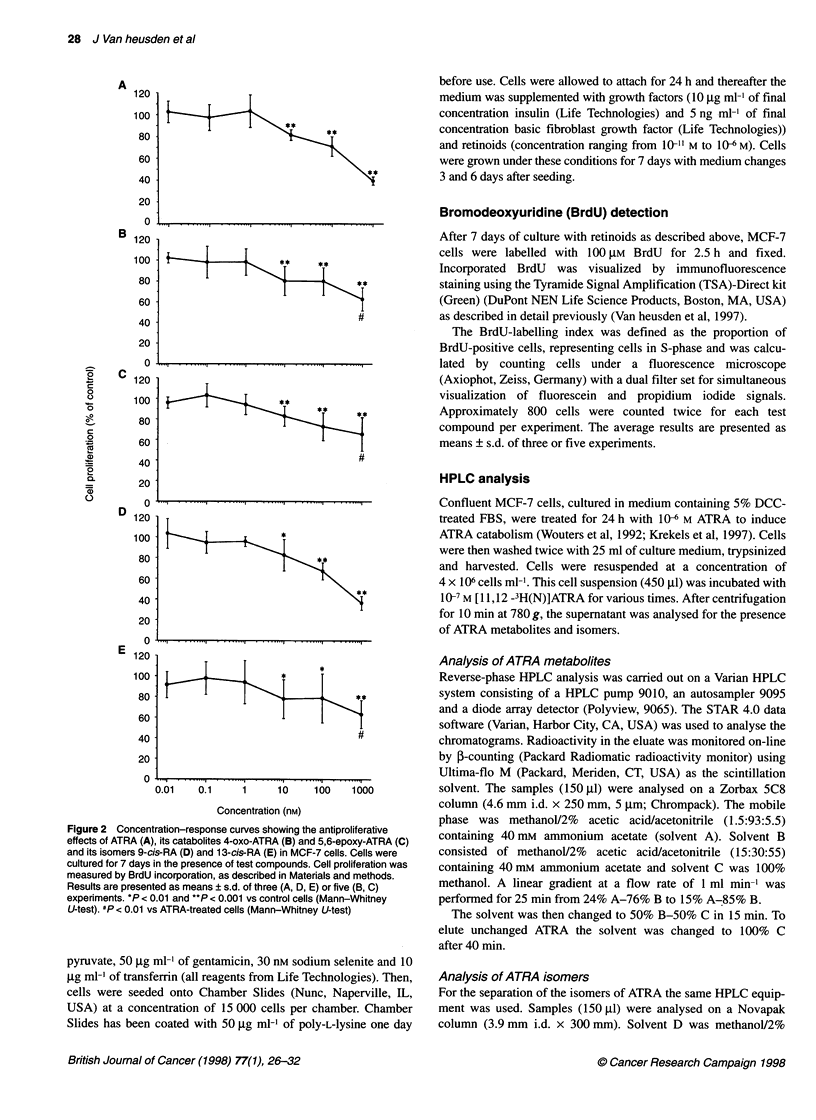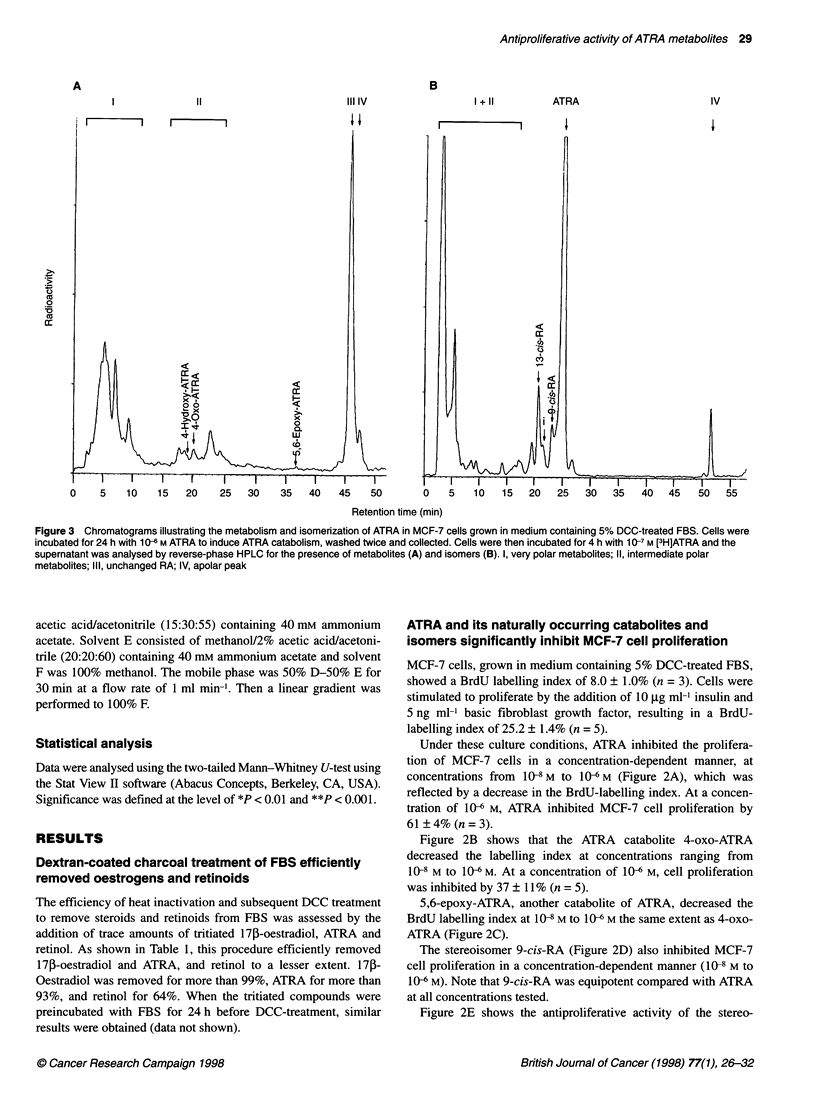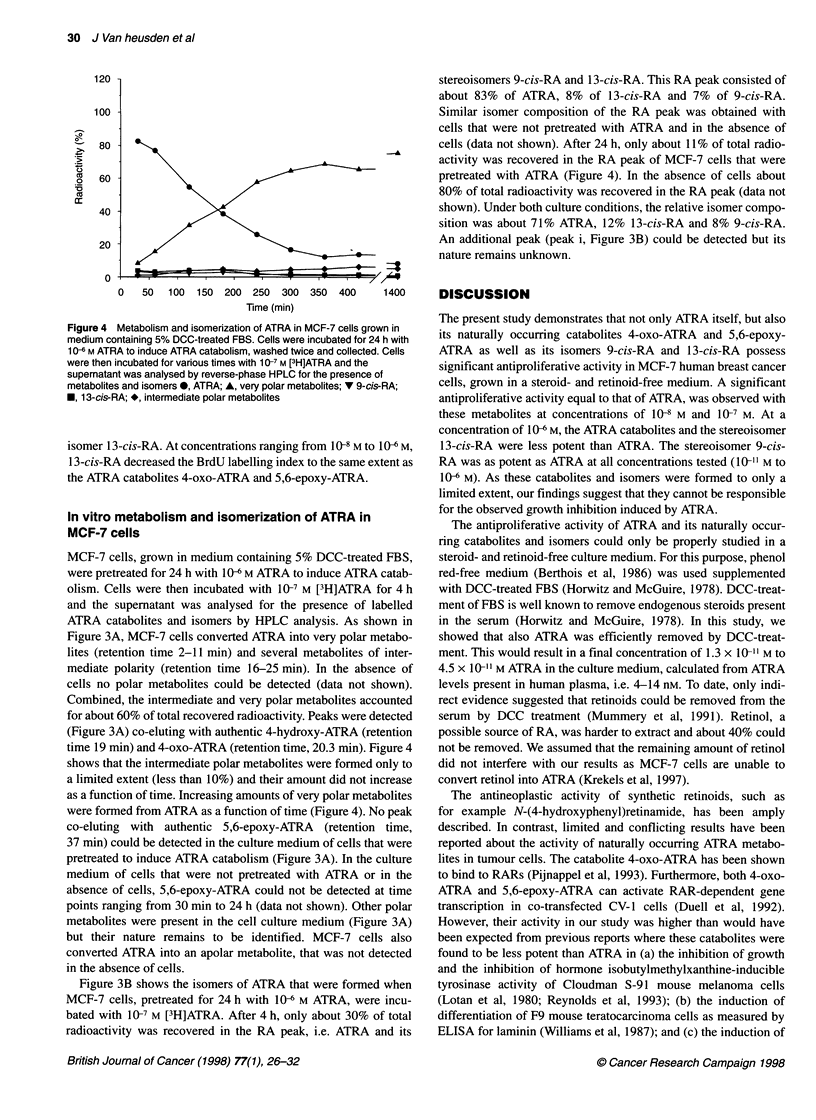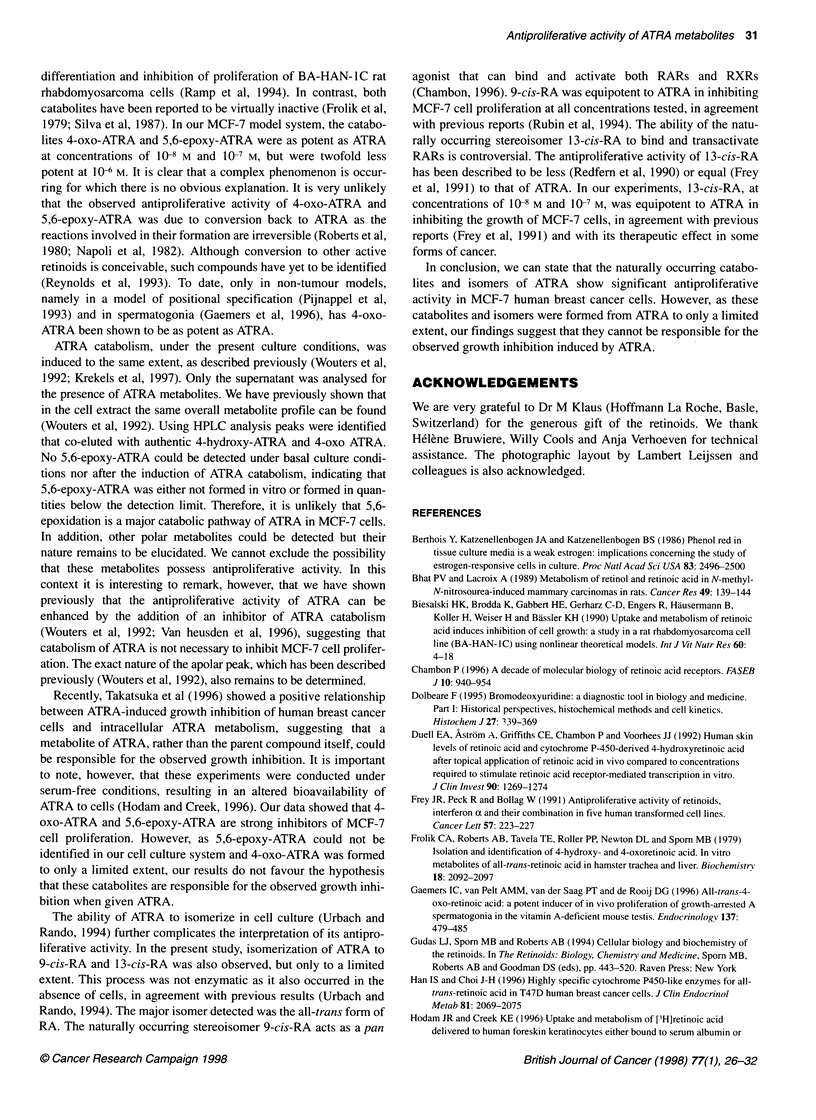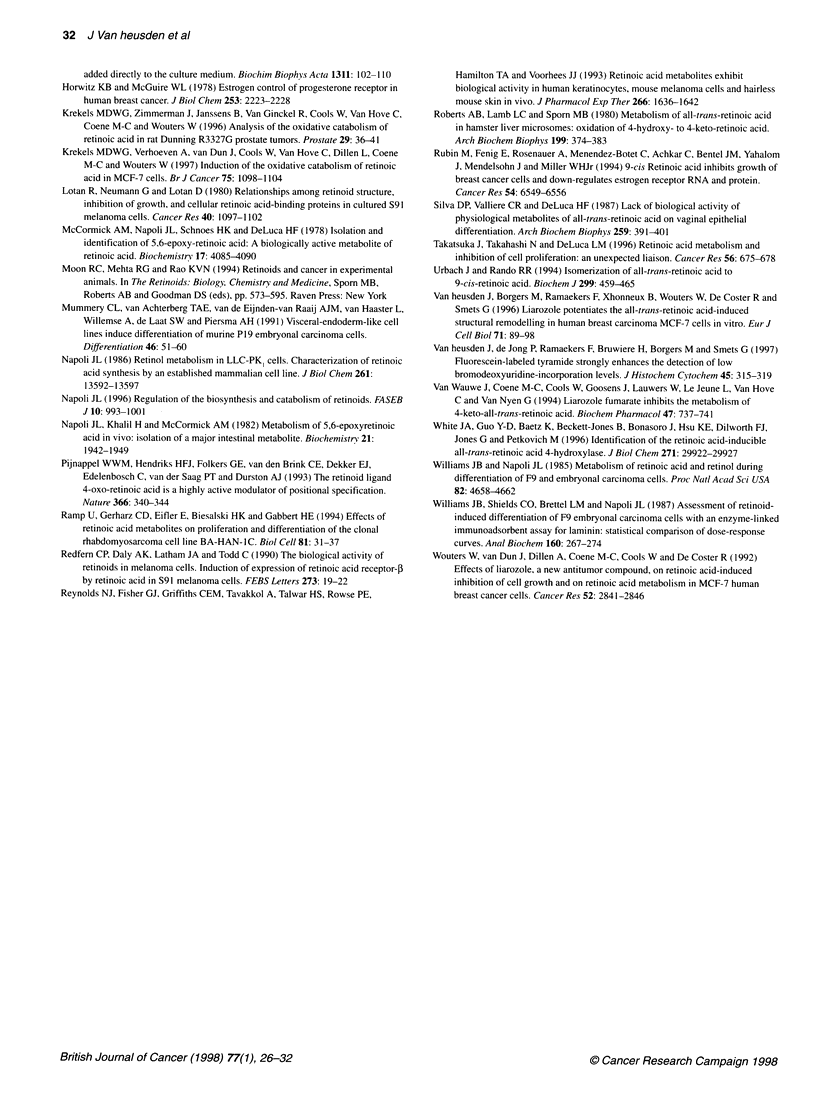# All-trans-retinoic acid metabolites significantly inhibit the proliferation of MCF-7 human breast cancer cells in vitro.

**DOI:** 10.1038/bjc.1998.5

**Published:** 1998

**Authors:** J. Van heusden, W. Wouters, F. C. Ramaekers, M. D. Krekels, L. Dillen, M. Borgers, G. Smets

**Affiliations:** Department of Molecular Cell Biology & Genetics, University of Maastricht, The Netherlands.

## Abstract

All-trans-retinoic acid (ATRA) is well known to inhibit the proliferation of human breast cancer cells. Much less is known about the antiproliferative activity of the naturally occurring metabolites and isomers of ATRA. In the present study, we investigated the antiproliferative activity of ATRA, its physiological catabolites 4-oxo-ATRA and 5,6-epoxy-ATRA and isomers 9-cis-RA and 13-cis-RA in MCF-7 human breast cancer cells by bromodeoxyuridine incorporation. MCF-7 cells were grown in steroid- and retinoid-free medium supplemented with growth factors. Under these culture conditions, ATRA and its naturally occurring catabolites and isomers showed significant antiproliferative activity in MCF-7 cells in a concentration-dependent manner (10[-11] M to 10[-6] M). The antiproliferative activity of ATRA catabolites and isomers was equal to that of the parent compound ATRA at concentrations of 10(-8) M and 10(-7) M. Only at 10(-6) M were the catabolites and the stereoisomer 13-cis-RA less potent. The stereoisomer 9-cis-RA was as potent as ATRA at all concentrations tested (10[-11] M to 10[-6] M). In addition, we show that the catabolites and isomers were formed from ATRA to only a limited extent. Together, our findings suggest that in spite of their high antiproliferative activity the catabolites and isomers of ATRA cannot be responsible for the observed growth inhibition induced by ATRA.


British Journal of Cancer (1998) 77(1), 26–32
© 1998 Cancer Research Campaign

### All-*trans*-retinoic acid metabolites significantly inhibit the proliferation of MCF-7 human breast cancer cells in vitro

**J Van heusden[1,2], W Wouters[2], FCS Ramaekers[1], MDWG Krekels[2], L Dillen[3], M Borgers[1,4] and G Smets[2]**

[1]Department of Molecular Cell Biology & Genetics, University of Maastricht, PO Box 616, 6200 MD Maastricht, The Netherlands; Departments of [2]Oncology, [3]Immunology and [4]Morphology, Janssen Research Foundation, Turnhoutseweg 30, B-2340 Beerse, Belgium

**Summary** All-*trans*-retinoic acid (ATRA) is well known to inhibit the proliferation of human breast cancer cells. Much less is known about the antiproliferative activity of the naturally occurring metabolites and isomers of ATRA. In the present study, we investigated the antiproliferative activity of ATRA, its physiological catabolites 4-oxo-ATRA and 5,6-epoxy-ATRA and isomers 9-*cis*-RA and 13-*cis*-RA in MCF-7 human breast cancer cells by bromodeoxyuridine incorporation. MCF-7 cells were grown in steroid- and retinoid-free medium supplemented with growth factors. Under these culture conditions, ATRA and its naturally occurring catabolites and isomers showed significant antiproliferative activity in MCF-7 cells in a concentration-dependent manner ($10^{-11}$ M to $10^{-6}$ M). The antiproliferative activity of ATRA catabolites and isomers was equal to that of the parent compound ATRA at concentrations of $10^{-8}$ M and $10^{-7}$ M. Only at $10^{-6}$ M were the catabolites and the stereoisomer 13-*cis*-RA less potent. The stereoisomer 9-*cis*-RA was as potent as ATRA at all concentrations tested ($10^{-11}$ M to $10^{-6}$ M). In addition, we show that the catabolites and isomers were formed from ATRA to only a limited extent. Together, our findings suggest that in spite of their high antiproliferative activity the catabolites and isomers of ATRA cannot be responsible for the observed growth inhibition induced by ATRA.

**Keywords:** retinoic acid; metabolism; breast cancer; MCF-7; catabolites; isomers

Retinoic acid (RA) has been shown to exert antiproliferative and differentiation-inducing effects on cancer cells both in vitro and in vivo (Gudas et al, 1994; Moon et al, 1994). These effects are mediated through binding to nuclear retinoid receptors, namely the RA receptors (RARs) and the retinoid X receptors (RXRs). Both receptors are members of the nuclear hormone receptor superfamily and function as ligand-dependent transcription factors (Chambon, 1996). The RAR family is activated both by ATRA and by 9-*cis*-RA, whereas the RXR family is activated exclusively by 9-*cis*-RA (Chambon, 1996). The ability of the natural stereoisomer 13-*cis*-RA to bind to RARs is controversial.

Diversity in the control of gene expression by RA exists because of the complexity at different levels of the signalling pathway. An important level in this complex retinoid signalling pathway is represented by the existence of natural metabolites of all-*trans*-RA (ATRA), whose synthesis may be modulated cell specifically (Napoli, 1996). ATRA metabolism has been studied in a variety of tissues and a number of metabolites have been identified (Figure 1). One important catabolic pathway for ATRA is initiated by hydroxylation at the four position of the β-ionone ring of ATRA to yield 4-hydroxy-ATRA (Frolik et al, 1979; Roberts et al, 1980). This step is catalysed by a cytochrome P450-dependent ATRA 4-hydroxylase (Roberts et al, 1980; White et al, 1996). 4-Hydroxy-ATRA is further oxidized via 4-oxo-ATRA to more polar metabolites (Roberts et al, 1980). This latter step involves at least one other, presently unknown, cytochrome P450-dependent enzyme (Roberts et al, 1980; Van Wauwe et al, 1994). Epoxidation of ATRA yields 5,6-epoxy-ATRA (McCormick et al, 1978). ATRA can also isomerize to 9-*cis*-RA and 13-*cis*-RA, an obviously non-enzymatic process (Urbach and Rando, 1994).

Cancer cells have been shown to possess RA catabolic activity. Oxidative catabolism of RA to more polar metabolites was observed in *N*-methyl-*N*-nitrosourea-induced mammary tumours in the rat (Bhat and Lacroix, 1989), in rat Dunning R3327G prostate tumours (Krekels et al, 1996), F9 mouse teratocarcinoma cells (Williams and Napoli, 1985), LLC-PK1 pig kidney cancer cells (Napoli, 1986), BA-HAN-1C rat rhabdomyosarcoma cells (Biesalski et al, 1990), as well as in the human breast cancer cells MCF-7 (Wouters et al, 1992; Krekels et al, 1997) and T47D (Han and Choi, 1996).

Recently, Takatsuka et al (1996) showed a positive relationship between metabolism of ATRA in human breast cancer cells and the antiproliferative activity of ATRA, suggesting that an undefined metabolite of ATRA, rather than the parent compound itself, could be responsible for the observed growth inhibition induced by ATRA. To date, only limited and conflicting results have been published concerning the activity of the naturally occurring ATRA metabolites in tumours. To clarify these points, we studied the antiproliferative activity of ATRA, its physiological catabolites 4-oxo-ATRA and 5,6-epoxy-ATRA and isomers 9-*cis*-RA and 13-*cis*-RA in MCF-7 cells using a bromodeoxyuridine incorporation assay. Bromodeoxyuridine labelling is considered an accurate method to measure cell proliferation because it is a direct assay of DNA synthesis (Dolbeare, 1995). Furthermore, the antiproliferative activities could only be properly studied in a steroid- and retinoid-free medium, because of the presence of endogenous retinoids in serum. Therefore, MCF-7 cells were grown in a phenol red-free medium supplemented with charcoal-treated fetal bovine serum and growth factors.

*Received 21 March 1997*
*Revised 9 June 1997*
*Accepted 12 June 1997*

*Correspondence to:* J Van heusden, Janssen Research Foundation, Department of Oncology, Turnhoutseweg 30, B-2340 Beerse, Belgium

**Figure 1** Chemical structures of ATRA, its naturally occurring isomers and catabolites arising from two different catabolic pathways

### MATERIALS AND METHODS

### Drugs and chemicals

ATRA was obtained from Serva (Heidelberg, Germany) and 13-*cis*-RA was purchased from Eastman Kodak (Rochester, NY, USA). 9-*cis*-RA, 4-oxo-ATRA and 5,6-epoxy-ATRA were a generous gift from Dr M Klaus (Hoffmann La Roche, Basle, Switzerland). All retinoids were dissolved in ethanol to an initial concentration of 4 mM and appropriately diluted in culture medium. The final solvent concentration during the proliferation studies was always ≤ 0.5% (v/v). The retinoid stock solutions were checked for purity using HPLC analysis. Experiments with retinoids were always performed in a dark room with yellow illumination.

### Preparation of dextran-coated charcoal (DCC)-treated fetal bovine serum (FBS)

DCC-treated FBS was prepared as described by Horwitz and McGuire (1978). Briefly, FBS (Life Technologies, Paisley, UK) was heat inactivated by a 30-min incubation at 56°C. Activated charcoal [0.25% (w/v); Sigma, St Louis, USA) was coated overnight at 4°C with dextran (0.025% (w/v); Pharmacia, Uppsala, Sweden) in 0.01 M Tris-HCl (pH 8.0). Then, 100 ml of this suspension was pelleted by centrifugation and 50 ml of heat-inactivated FBS was incubated with the resulting DCC pellet for 45 min at 45°C. This procedure was repeated, and finally the activated charcoal was removed from the FBS by centrifugation. DCC-treated FBS was sterilized by passage through a 0.22 μm Millipore filter (low protein binding) and stored at –20°C until use. The efficiency of this procedure was assessed by the addition of a trace amount of [6,7-$^3$H(N)]oestradiol (Dupont NEN, Boston, MA, USA), [11,12-$^3$H(N)]retinol (Dupont NEN, Boston, MA, USA) and [11,12-$^3$H(N)]ATRA (Dupont NEN, Boston, MA, USA).

**Table 1** Efficiency of oestrogen and retinoid extraction from fetal bovine serum by dextran-coated charcoal treatment

| | Amount of tritiated label after DCC treatment (%) |
|---|---|
| 17β-Oestradiol | 0.64 ± 0.17 |
| ATRA | 6.43 ± 0.52 |
| Retinol | 35.78 ± 6.78 |

Mean ± s.d. ($n$ = 3).

### Cell culture

Stock cultures of MCF-7 human breast cancer cells, purchased from the American Type Culture Collection (Rockville, MD, USA), were cultured in Dulbecco's modified Eagle medium (DMEM) with 4.5 g $l^{-1}$ of glucose supplemented with 10% (v/v) FBS, 2 mM L-glutamine, 1 mM sodium pyruvate and 50 μg $ml^{-1}$ gentamicin (all reagents from Life Technologies). The MCF-7 subclone used in this study has been characterized previously (Van heusden et al, 1996). Cells were grown in a humidified incubator (5% carbon dioxide, 95% air) at 37°C and were *Mycoplasma*-free as tested by the *Mycoplasma* TC kit (Gen-Probe Incorporated, CA, USA).

For the proliferation studies, the MCF-7 cells were cultured for 6 days in phenol red-free DMEM containing 5% (v/v) DCC-treated FBS, 4.5 g $l^{-1}$ of glucose, 2 mM L-glutamine, 1 mM sodium

**Figure 2** Concentration–response curves showing the antiproliferative effects of ATRA (**A**), its catabolites 4-oxo-ATRA (**B**) and 5,6-epoxy-ATRA (**C**) and its isomers 9-*cis*-RA (**D**) and 13-*cis*-RA (**E**) in MCF-7 cells. Cells were cultured for 7 days in the presence of test compounds. Cell proliferation was measured by BrdU incorporation, as described in Materials and methods. Results are presented as means ± s.d. of three (A, D, E) or five (B, C) experiments. $^*P < 0.01$ and $^{**}P < 0.001$ vs control cells (Mann–Whitney *U*-test). $^{\#}P < 0.01$ vs ATRA-treated cells (Mann–Whitney *U*-test)

pyruvate, 50 μg $ml^{-1}$ of gentamicin, 30 nM sodium selenite and 10 μg $ml^{-1}$ of transferrin (all reagents from Life Technologies). Then, cells were seeded onto Chamber Slides (Nunc, Naperville, IL, USA) at a concentration of 15 000 cells per chamber. Chamber Slides has been coated with 50 μg $ml^{-1}$ of poly-L-lysine one day before use. Cells were allowed to attach for 24 h and thereafter the medium was supplemented with growth factors (10 μg $ml^{-1}$ of final concentration insulin (Life Technologies) and 5 ng $ml^{-1}$ of final concentration basic fibroblast growth factor (Life Technologies)) and retinoids (concentration ranging from $10^{-11}$ M to $10^{-6}$ M). Cells were grown under these conditions for 7 days with medium changes 3 and 6 days after seeding.

### Bromodeoxyuridine (BrdU) detection

After 7 days of culture with retinoids as described above, MCF-7 cells were labelled with 100 μM BrdU for 2.5 h and fixed. Incorporated BrdU was visualized by immunofluorescence staining using the Tyramide Signal Amplification (TSA)-Direct kit (Green) (DuPont NEN Life Science Products, Boston, MA, USA) as described in detail previously (Van heusden et al, 1997).

The BrdU-labelling index was defined as the proportion of BrdU-positive cells, representing cells in S-phase and was calculated by counting cells under a fluorescence microscope (Axiophot, Zeiss, Germany) with a dual filter set for simultaneous visualization of fluorescein and propidium iodide signals. Approximately 800 cells were counted twice for each test compound per experiment. The average results are presented as means ± s.d. of three or five experiments.

### HPLC analysis

Confluent MCF-7 cells, cultured in medium containing 5% DCC-treated FBS, were treated for 24 h with $10^{-6}$ M ATRA to induce ATRA catabolism (Wouters et al, 1992; Krekels et al, 1997). Cells were then washed twice with 25 ml of culture medium, trypsinized and harvested. Cells were resuspended at a concentration of $4 \times 10^6$ cells $ml^{-1}$. This cell suspension (450 μl) was incubated with $10^{-7}$ M [11,12 -$^3$H(N)]ATRA for various times. After centrifugation for 10 min at 780 *g*, the supernatant was analysed for the presence of ATRA metabolites and isomers.

### *Analysis of ATRA metabolites*

Reverse-phase HPLC analysis was carried out on a Varian HPLC system consisting of a HPLC pump 9010, an autosampler 9095 and a diode array detector (Polyview, 9065). The STAR 4.0 data software (Varian, Harbor City, CA, USA) was used to analyse the chromatograms. Radioactivity in the eluate was monitored on-line by β-counting (Packard Radiomatic radioactivity monitor) using Ultima-flo M (Packard, Meriden, CT, USA) as the scintillation solvent. The samples (150 μl) were analysed on a Zorbax 5C8 column (4.6 mm i.d. × 250 mm, 5 μm; Chrompack). The mobile phase was methanol/2% acetic acid/acetonitrile (1.5:93:5.5) containing 40 mM ammonium acetate (solvent A). Solvent B consisted of methanol/2% acetic acid/acetonitrile (15:30:55) containing 40 mM ammonium acetate and solvent C was 100% methanol. A linear gradient at a flow rate of 1 ml $min^{-1}$ was performed for 25 min from 24% A–76% B to 15% A–85% B.

The solvent was then changed to 50% B–50% C in 15 min. To elute unchanged ATRA the solvent was changed to 100% C after 40 min.

### *Analysis of ATRA isomers*

For the separation of the isomers of ATRA the same HPLC equipment was used. Samples (150 μl) were analysed on a Novapak column (3.9 mm i.d. × 300 mm). Solvent D was methanol/2%

© Cancer Research Campaign 1998

**Figure 3** Chromatograms illustrating the metabolism and isomerization of ATRA in MCF-7 cells grown in medium containing 5% DCC-treated FBS. Cells were incubated for 24 h with $10^{-6}$ M ATRA to induce ATRA catabolism, washed twice and collected. Cells were then incubated for 4 h with $10^{-7}$ M [$^3$H]ATRA and the supernatant was analysed by reverse-phase HPLC for the presence of metabolites (**A**) and isomers (**B**). I, very polar metabolites; II, intermediate polar metabolites; III, unchanged RA; IV, apolar peak acetic acid/acetonitrile (15:30:55) containing 40 mM ammonium acetate. Solvent E consisted of methanol/2% acetic acid/acetonitrile (20:20:60) containing 40 mM ammonium acetate and solvent F was 100% methanol. The mobile phase was 50% D–50% E for 30 min at a flow rate of 1 ml $min^{-1}$. Then a linear gradient was performed to 100% F.

### Statistical analysis

Data were analysed using the two-tailed Mann–Whitney *U*-test using the Stat View II software (Abacus Concepts, Berkeley, CA, USA). Significance was defined at the level of $^*P < 0.01$ and $^{**}P < 0.001$.

### RESULTS

### Dextran-coated charcoal treatment of FBS efficiently removed oestrogens and retinoids

The efficiency of heat inactivation and subsequent DCC treatment to remove steroids and retinoids from FBS was assessed by the addition of trace amounts of tritiated 17β-oestradiol, ATRA and retinol. As shown in Table 1, this procedure efficiently removed 17β-oestradiol and ATRA, and retinol to a lesser extent. 17β-Oestradiol was removed for more than 99%, ATRA for more than 93%, and retinol for 64%. When the tritiated compounds were preincubated with FBS for 24 h before DCC-treatment, similar results were obtained (data not shown).

### ATRA and its naturally occurring catabolites and isomers significantly inhibit MCF-7 cell proliferation

MCF-7 cells, grown in medium containing 5% DCC-treated FBS, showed a BrdU labelling index of 8.0 ± 1.0% ($n = 3$). Cells were stimulated to proliferate by the addition of 10 µg $ml^{-1}$ insulin and 5 ng $ml^{-1}$ basic fibroblast growth factor, resulting in a BrdU-labelling index of 25.2 ± 1.4% ($n = 5$).

Under these culture conditions, ATRA inhibited the proliferation of MCF-7 cells in a concentration-dependent manner, at concentrations from $10^{-8}$ M to $10^{-6}$ M (Figure 2A), which was reflected by a decrease in the BrdU-labelling index. At a concentration of $10^{-6}$ M, ATRA inhibited MCF-7 cell proliferation by 61 ± 4% ($n = 3$).

Figure 2B shows that the ATRA catabolite 4-oxo-ATRA decreased the labelling index at concentrations ranging from $10^{-8}$ M to $10^{-6}$ M. At a concentration of $10^{-6}$ M, cell proliferation was inhibited by 37 ± 11% ($n = 5$).

5,6-epoxy-ATRA, another catabolite of ATRA, decreased the BrdU labelling index at $10^{-8}$ M to $10^{-6}$ M the same extent as 4-oxo-ATRA (Figure 2C).

The stereoisomer 9-*cis*-RA (Figure 2D) also inhibited MCF-7 cell proliferation in a concentration-dependent manner ($10^{-8}$ M to $10^{-6}$ M). Note that 9-*cis*-RA was equipotent compared with ATRA at all concentrations tested.

Figure 2E shows the antiproliferative activity of the stereo-

© Cancer Research Campaign 1998

**Figure 4** Metabolism and isomerization of ATRA in MCF-7 cells grown in medium containing 5% DCC-treated FBS. Cells were incubated for 24 h with $10^{-6}$ M ATRA to induce ATRA catabolism, washed twice and collected. Cells were then incubated for various times with $10^{-7}$ M [$^3$H]ATRA and the supernatant was analysed by reverse-phase HPLC for the presence of metabolites and isomers ●, ATRA; ▲, very polar metabolites; ▼ 9-*cis*-RA; ■, 13-*cis*-RA; ◆, intermediate polar metabolites isomer 13-*cis*-RA. At concentrations ranging from $10^{-8}$ M to $10^{-6}$ M, 13-*cis*-RA decreased the BrdU labelling index to the same extent as the ATRA catabolites 4-oxo-ATRA and 5,6-epoxy-ATRA.

### In vitro metabolism and isomerization of ATRA in MCF-7 cells

MCF-7 cells, grown in medium containing 5% DCC-treated FBS, were pretreated for 24 h with $10^{-6}$ M ATRA to induce ATRA catabolism. Cells were then incubated with $10^{-7}$ M [$^3$H]ATRA for 4 h and the supernatant was analysed for the presence of labelled ATRA catabolites and isomers by HPLC analysis. As shown in Figure 3A, MCF-7 cells converted ATRA into very polar metabolites (retention time 2–11 min) and several metabolites of intermediate polarity (retention time 16–25 min). In the absence of cells no polar metabolites could be detected (data not shown). Combined, the intermediate and very polar metabolites accounted for about 60% of total recovered radioactivity. Peaks were detected (Figure 3A) co-eluting with authentic 4-hydroxy-ATRA (retention time 19 min) and 4-oxo-ATRA (retention time, 20.3 min). Figure 4 shows that the intermediate polar metabolites were formed only to a limited extent (less than 10%) and their amount did not increase as a function of time. Increasing amounts of very polar metabolites were formed from ATRA as a function of time (Figure 4). No peak co-eluting with authentic 5,6-epoxy-ATRA (retention time, 37 min) could be detected in the culture medium of cells that were pretreated to induce ATRA catabolism (Figure 3A). In the culture medium of cells that were not pretreated with ATRA or in the absence of cells, 5,6-epoxy-ATRA could not be detected at time points ranging from 30 min to 24 h (data not shown). Other polar metabolites were present in the cell culture medium (Figure 3A) but their nature remains to be identified. MCF-7 cells also converted ATRA into an apolar metabolite, that was not detected in the absence of cells.

Figure 3B shows the isomers of ATRA that were formed when MCF-7 cells, pretreated for 24 h with $10^{-6}$ M ATRA, were incubated with $10^{-7}$ M [$^3$H]ATRA. After 4 h, only about 30% of total radioactivity was recovered in the RA peak, i.e. ATRA and its stereoisomers 9-*cis*-RA and 13-*cis*-RA. This RA peak consisted of about 83% of ATRA, 8% of 13-*cis*-RA and 7% of 9-*cis*-RA. Similar isomer composition of the RA peak was obtained with cells that were not pretreated with ATRA and in the absence of cells (data not shown). After 24 h, only about 11% of total radioactivity was recovered in the RA peak of MCF-7 cells that were pretreated with ATRA (Figure 4). In the absence of cells about 80% of total radioactivity was recovered in the RA peak (data not shown). Under both culture conditions, the relative isomer composition was about 71% ATRA, 12% 13-*cis*-RA and 8% 9-*cis*-RA. An additional peak (peak i, Figure 3B) could be detected but its nature remains unknown.

### DISCUSSION

The present study demonstrates that not only ATRA itself, but also its naturally occurring catabolites 4-oxo-ATRA and 5,6-epoxy-ATRA as well as its isomers 9-*cis*-RA and 13-*cis*-RA possess significant antiproliferative activity in MCF-7 human breast cancer cells, grown in a steroid- and retinoid-free medium. A significant antiproliferative activity equal to that of ATRA, was observed with these metabolites at concentrations of $10^{-8}$ M and $10^{-7}$ M. At a concentration of $10^{-6}$ M, the ATRA catabolites and the stereoisomer 13-*cis*-RA were less potent than ATRA. The stereoisomer 9-*cis*-RA was as potent as ATRA at all concentrations tested ($10^{-11}$ M to $10^{-6}$ M). As these catabolites and isomers were formed to only a limited extent, our findings suggest that they cannot be responsible for the observed growth inhibition induced by ATRA.

The antiproliferative activity of ATRA and its naturally occurring catabolites and isomers could only be properly studied in a steroid- and retinoid-free culture medium. For this purpose, phenol red-free medium (Berthois et al, 1986) was used supplemented with DCC-treated FBS (Horwitz and McGuire, 1978). DCC-treatment of FBS is well known to remove endogenous steroids present in the serum (Horwitz and McGuire, 1978). In this study, we showed that also ATRA was efficiently removed by DCC-treatment. This would result in a final concentration of $1.3 \times 10^{-11}$ M to $4.5 \times 10^{-11}$ M ATRA in the culture medium, calculated from ATRA levels present in human plasma, i.e. 4–14 nM. To date, only indirect evidence suggested that retinoids could be removed from the serum by DCC treatment (Mummery et al, 1991). Retinol, a possible source of RA, was harder to extract and about 40% could not be removed. We assumed that the remaining amount of retinol did not interfere with our results as MCF-7 cells are unable to convert retinol into ATRA (Krekels et al, 1997).

The antineoplastic activity of synthetic retinoids, such as for example *N*-(4-hydroxyphenyl)retinamide, has been amply described. In contrast, limited and conflicting results have been reported about the activity of naturally occurring ATRA metabolites in tumour cells. The catabolite 4-oxo-ATRA has been shown to bind to RARs (Pijnappel et al, 1993). Furthermore, both 4-oxo-ATRA and 5,6-epoxy-ATRA can activate RAR-dependent gene transcription in co-transfected CV-1 cells (Duell et al, 1992). However, their activity in our study was higher than would have been expected from previous reports where these catabolites were found to be less potent than ATRA in (a) the inhibition of growth and the inhibition of hormone isobutylmethylxanthine-inducible tyrosinase activity of Cloudman S-91 mouse melanoma cells (Lotan et al, 1980; Reynolds et al, 1993); (b) the induction of differentiation of F9 mouse teratocarcinoma cells as measured by ELISA for laminin (Williams et al, 1987); and (c) the induction of

© Cancer Research Campaign 1998

differentiation and inhibition of proliferation of BA-HAN-1C rat rhabdomyosarcoma cells (Ramp et al, 1994). In contrast, both catabolites have been reported to be virtually inactive (Frolik et al, 1979; Silva et al, 1987). In our MCF-7 model system, the catabolites 4-oxo-ATRA and 5,6-epoxy-ATRA were as potent as ATRA at concentrations of $10^{-8}$ M and $10^{-7}$ M, but were twofold less potent at $10^{-6}$ M. It is clear that a complex phenomenon is occurring for which there is no obvious explanation. It is very unlikely that the observed antiproliferative activity of 4-oxo-ATRA and 5,6-epoxy-ATRA was due to conversion back to ATRA as the reactions involved in their formation are irreversible (Roberts et al, 1980; Napoli et al, 1982). Although conversion to other active retinoids is conceivable, such compounds have yet to be identified (Reynolds et al, 1993). To date, only in non-tumour models, namely in a model of positional specification (Pijnappel et al, 1993) and in spermatogonia (Gaemers et al, 1996), has 4-oxo-ATRA been shown to be as potent as ATRA.

ATRA catabolism, under the present culture conditions, was induced to the same extent, as described previously (Wouters et al, 1992; Krekels et al, 1997). Only the supernatant was analysed for the presence of ATRA metabolites. We have previously shown that in the cell extract the same overall metabolite profile can be found (Wouters et al, 1992). Using HPLC analysis peaks were identified that co-eluted with authentic 4-hydroxy-ATRA and 4-oxo ATRA. No 5,6-epoxy-ATRA could be detected under basal culture conditions nor after the induction of ATRA catabolism, indicating that 5,6-epoxy-ATRA was either not formed in vitro or formed in quantities below the detection limit. Therefore, it is unlikely that 5,6-epoxidation is a major catabolic pathway of ATRA in MCF-7 cells. In addition, other polar metabolites could be detected but their nature remains to be elucidated. We cannot exclude the possibility that these metabolites possess antiproliferative activity. In this context it is interesting to remark, however, that we have shown previously that the antiproliferative activity of ATRA can be enhanced by the addition of an inhibitor of ATRA catabolism (Wouters et al, 1992; Van heusden et al, 1996), suggesting that catabolism of ATRA is not necessary to inhibit MCF-7 cell proliferation. The exact nature of the apolar peak, which has been described previously (Wouters et al, 1992), also remains to be determined.

Recently, Takatsuka et al (1996) showed a positive relationship between ATRA-induced growth inhibition of human breast cancer cells and intracellular ATRA metabolism, suggesting that a metabolite of ATRA, rather than the parent compound itself, could be responsible for the observed growth inhibition. It is important to note, however, that these experiments were conducted under serum-free conditions, resulting in an altered bioavailability of ATRA to cells (Hodam and Creek, 1996). Our data showed that 4-oxo-ATRA and 5,6-epoxy-ATRA are strong inhibitors of MCF-7 cell proliferation. However, as 5,6-epoxy-ATRA could not be identified in our cell culture system and 4-oxo-ATRA was formed to only a limited extent, our results do not favour the hypothesis that these catabolites are responsible for the observed growth inhibition when given ATRA.

The ability of ATRA to isomerize in cell culture (Urbach and Rando, 1994) further complicates the interpretation of its antiproliferative activity. In the present study, isomerization of ATRA to 9-*cis*-RA and 13-*cis*-RA was also observed, but only to a limited extent. This process was not enzymatic as it also occurred in the absence of cells, in agreement with previous results (Urbach and Rando, 1994). The major isomer detected was the all-*trans* form of RA. The naturally occurring stereoisomer 9-*cis*-RA acts as a *pan* agonist that can bind and activate both RARs and RXRs (Chambon, 1996). 9-*cis*-RA was equipotent to ATRA in inhibiting MCF-7 cell proliferation at all concentrations tested, in agreement with previous reports (Rubin et al, 1994). The ability of the naturally occurring stereoisomer 13-*cis*-RA to bind and transactivate RARs is controversial. The antiproliferative activity of 13-*cis*-RA has been described to be less (Redfern et al, 1990) or equal (Frey et al, 1991) to that of ATRA. In our experiments, 13-*cis*-RA, at concentrations of $10^{-8}$ M and $10^{-7}$ M, was equipotent to ATRA in inhibiting the growth of MCF-7 cells, in agreement with previous reports (Frey et al, 1991) and with its therapeutic effect in some forms of cancer.

In conclusion, we can state that the naturally occurring catabolites and isomers of ATRA show significant antiproliferative activity in MCF-7 human breast cancer cells. However, as these catabolites and isomers were formed from ATRA to only a limited extent, our findings suggest that they cannot be responsible for the observed growth inhibition induced by ATRA.

### ACKNOWLEDGEMENTS

We are very grateful to Dr M Klaus (Hoffmann La Roche, Basle, Switzerland) for the generous gift of the retinoids. We thank Hélène Bruwiere, Willy Cools and Anja Verhoeven for technical assistance. The photographic layout by Lambert Leijssen and colleagues is also acknowledged.

### REFERENCES

Berthois Y, Katzenellenbogen JA and Katzenellenbogen BS (1986) Phenol red in tissue culture media is a weak estrogen: implications concerning the study of estrogen-responsive cells in culture. *Proc Natl Acad Sci USA* **83**: 2496–2500

Bhat PV and Lacroix A (1989) Metabolism of retinol and retinoic acid in *N*-methyl-*N*-nitrosourea-induced mammary carcinomas in rats. *Cancer Res* **49**: 139–144

Biesalski HK, Brodda K, Gabbert HE, Gerharz C-D, Engers R, Häusermann B, Koller H, Weiser H and Bässler KH (1990) Uptake and metabolism of retinoic acid induces inhibition of cell growth: a study in a rat rhabdomyosarcoma cell line (BA-HAN-1C) using nonlinear theoretical models. *Int J Vit Nutr Res* **60**: 4–18

Chambon P (1996) A decade of molecular biology of retinoic acid receptors. *FASEB J* **10**: 940–954

Dolbeare F (1995) Bromodeoxyuridine: a diagnostic tool in biology and medicine. Part I: Historical perspectives, histochemical methods and cell kinetics. *Histochem J* **27**: 339–369

Duell EA, Åström A, Griffiths CE, Chambon P and Voorhees JJ (1992) Human skin levels of retinoic acid and cytochrome P-450-derived 4-hydroxyretinoic acid after topical application of retinoic acid in vivo compared to concentrations required to stimulate retinoic acid receptor-mediated transcription in vitro. *J Clin Invest* **90**: 1269–1274

Frey JR, Peck R and Bollag W (1991) Antiproliferative activity of retinoids, interferon α and their combination in five human transformed cell lines. *Cancer Lett* **57**: 223–227

Frolik CA, Roberts AB, Tavela TE, Roller PP, Newton DL and Sporn MB (1979) Isolation and identification of 4-hydroxy- and 4-oxoretinoic acid. In vitro metabolites of all-*trans*-retinoic acid in hamster trachea and liver. *Biochemistry* **18**: 2092–2097

Gaemers IC, van Pelt AMM, van der Saag PT and de Rooij DG (1996) All-*trans*-4-oxo-retinoic acid: a potent inducer of in vivo proliferation of growth-arrested A spermatogonia in the vitamin A-deficient mouse testis. *Endocrinology* **137**: 479–485

Gudas LJ, Sporn MB and Roberts AB (1994) Cellular biology and biochemistry of the retinoids. In *The Retinoids: Biology, Chemistry and Medicine*, Sporn MB, Roberts AB and Goodman DS (eds), pp. 443–520. Raven Press: New York

Han IS and Choi J-H (1996) Highly specific cytochrome P450-like enzymes for all-*trans*-retinoic acid in T47D human breast cancer cells. *J Clin Endocrinol Metab* **81**: 2069–2075

Hodam JR and Creek KE (1996) Uptake and metabolism of [$^3$H]retinoic acid delivered to human foreskin keratinocytes either bound to serum albumin or added directly to the culture medium. *Biochim Biophys Acta* **1311**: 102–110

Horwitz KB and McGuire WL (1978) Estrogen control of progesterone receptor in human breast cancer. *J Biol Chem* **253**: 2223–2228

Krekels MDWG, Zimmerman J, Janssens B, Van Ginckel R, Cools W, Van Hove C, Coene M-C and Wouters W (1996) Analysis of the oxidative catabolism of retinoic acid in rat Dunning R3327G prostate tumors. *Prostate* **29**: 36–41

Krekels MDWG, Verhoeven A, van Dun J, Cools W, Van Hove C, Dillen L, Coene M-C and Wouters W (1997) Induction of the oxidative catabolism of retinoic acid in MCF-7 cells. *Br J Cancer* **75**: 1098–1104

Lotan R, Neumann G and Lotan D (1980) Relationships among retinoid structure, inhibition of growth, and cellular retinoic acid-binding proteins in cultured S91 melanoma cells. *Cancer Res* **40**: 1097–1102

McCormick AM, Napoli JL, Schnoes HK and DeLuca HF (1978) Isolation and identification of 5,6-epoxy-retinoic acid: A biologically active metabolite of retinoic acid. *Biochemistry* **17**: 4085–4090

Moon RC, Mehta RG and Rao KVN (1994) Retinoids and cancer in experimental animals. In *The Retinoids: Biology, Chemistry and Medicine*, Sporn MB, Roberts AB and Goodman DS (eds), pp. 573–595. Raven Press: New York

Mummery CL, van Achterberg TAE, van de Eijnden-van Raaij AJM, van Haaster L, Willemse A, de Laat SW and Piersma AH (1991) Visceral-endoderm-like cell lines induce differentiation of murine P19 embryonal carcinoma cells. *Differentiation* **46**: 51–60

Napoli JL (1986) Retinol metabolism in LLC-$PK_1$ cells. Characterization of retinoic acid synthesis by an established mammalian cell line. *J Biol Chem* **261**: 13592–13597

Napoli JL (1996) Regulation of the biosynthesis and catabolism of retinoids. *FASEB J* **10**: 993–1001

Napoli JL, Khalil H and McCormick AM (1982) Metabolism of 5,6-epoxyretinoic acid in vivo: isolation of a major intestinal metabolite. *Biochemistry* **21**: 1942–1949

Pijnappel WWM, Hendriks HFJ, Folkers GE, van den Brink CE, Dekker EJ, Edelenbosch C, van der Saag PT and Durston AJ (1993) The retinoid ligand 4-oxo-retinoic acid is a highly active modulator of positional specification. *Nature* **366**: 340–344

Ramp U, Gerharz CD, Eifler E, Biesalski HK and Gabbert HE (1994) Effects of retinoic acid metabolites on proliferation and differentiation of the clonal rhabdomyosarcoma cell line BA-HAN-1C. *Biol Cell* **81**: 31–37

Redfern CP, Daly AK, Latham JA and Todd C (1990) The biological activity of retinoids in melanoma cells. Induction of expression of retinoic acid receptor-β by retinoic acid in S91 melanoma cells. *FEBS Letters* **273**: 19–22

Reynolds NJ, Fisher GJ, Griffiths CEM, Tavakkol A, Talwar HS, Rowse PE, Hamilton TA and Voorhees JJ (1993) Retinoic acid metabolites exhibit biological activity in human keratinocytes, mouse melanoma cells and hairless mouse skin in vivo. *J Pharmacol Exp Ther* **266**: 1636–1642

Roberts AB, Lamb LC and Sporn MB (1980) Metabolism of all-*trans*-retinoic acid in hamster liver microsomes: oxidation of 4-hydroxy- to 4-keto-retinoic acid. *Arch Biochem Biophys* **199**: 374–383

Rubin M, Fenig E, Rosenauer A, Menendez-Botet C, Achkar C, Bentel JM, Yahalom J, Mendelsohn J and Miller WHJr (1994) 9-*cis* Retinoic acid inhibits growth of breast cancer cells and down-regulates estrogen receptor RNA and protein. *Cancer Res* **54**: 6549–6556

Silva DP, Valliere CR and DeLuca HF (1987) Lack of biological activity of physiological metabolites of all-*trans*-retinoic acid on vaginal epithelial differentiation. *Arch Biochem Biophys* **259**: 391–401

Takatsuka J, Takahashi N and DeLuca LM (1996) Retinoic acid metabolism and inhibition of cell proliferation: an unexpected liaison. *Cancer Res* **56**: 675–678

Urbach J and Rando RR (1994) Isomerization of all-*trans*-retinoic acid to 9-*cis*-retinoic acid. *Biochem J* **299**: 459–465

Van heusden J, Borgers M, Ramaekers F, Xhonneux B, Wouters W, De Coster R and Smets G (1996) Liarozole potentiates the all-*trans*-retinoic acid-induced structural remodelling in human breast carcinoma MCF-7 cells in vitro. *Eur J Cell Biol* **71**: 89–98

Van heusden J, de Jong P, Ramaekers F, Bruwiere H, Borgers M and Smets G (1997) Fluorescein-labeled tyramide strongly enhances the detection of low bromodeoxyuridine-incorporation levels. *J Histochem Cytochem* **45**: 315–319

Van Wauwe J, Coene M-C, Cools W, Goosens J, Lauwers W, Le Jeune L, Van Hove C and Van Nyen G (1994) Liarozole fumarate inhibits the metabolism of 4-keto-all-*trans*-retinoic acid. *Biochem Pharmacol* **47**: 737–741

White JA, Guo Y-D, Baetz K, Beckett-Jones B, Bonasoro J, Hsu KE, Dilworth FJ, Jones G and Petkovich M (1996) Identification of the retinoic acid-inducible all-*trans*-retinoic acid 4-hydroxylase. *J Biol Chem* **271**: 29922–29927

Williams JB and Napoli JL (1985) Metabolism of retinoic acid and retinol during differentiation of F9 and embryonal carcinoma cells. *Proc Natl Acad Sci USA* **82**: 4658–4662

Williams JB, Shields CO, Brettel LM and Napoli JL (1987) Assessment of retinoid-induced differentiation of F9 embryonal carcinoma cells with an enzyme-linked immunoadsorbent assay for laminin: statistical comparison of dose-response curves. *Anal Biochem* **160**: 267–274

Wouters W, van Dun J, Dillen A, Coene M-C, Cools W and De Coster R (1992) Effects of liarozole, a new antitumor compound, on retinoic acid-induced inhibition of cell growth and on retinoic acid metabolism in MCF-7 human breast cancer cells. *Cancer Res* **52**: 2841–2846

© Cancer Research Campaign 1998